# Caregiver’s quality of life in advanced cancer: validation of the construct in a real-life setting of early palliative care

**DOI:** 10.3389/fonc.2023.1213906

**Published:** 2023-09-14

**Authors:** Eleonora Borelli, Sarah Bigi, Leonardo Potenza, Fabio Gilioli, Fabio Efficace, Carlo Adolfo Porro, Mario Luppi, Elena Bandieri

**Affiliations:** ^1^ Department of Medical and Surgical Sciences, University of Modena and Reggio Emilia, Modena, Italy; ^2^ Department of Linguistic Sciences and Foreign Literatures, Catholic University of the Sacred Heart, Milan, Italy; ^3^ Hematology Unit and Chair, Azienda Ospedaliera Universitaria di Modena, Modena, Italy; ^4^ Department of Internal Medicine and Rehabilitation, Unità Sanitaria Locale (USL), Modena, Italy; ^5^ Health Outcomes Research Unit, Italian Group for Adult Hematologic Diseases (GIMEMA), Rome, Italy; ^6^ Department of Biomedical, Metabolic and Neural Sciences, University of Modena and Reggio Emilia, Modena, Italy; ^7^ Center for Neuroscience and Neurotechnology, University of Modena and Reggio Emilia, Modena, Italy; ^8^ Oncology and Palliative Care Units, Civil Hospital Carpi, Unità Sanitaria Locale (USL), Carpi, Italy

**Keywords:** early palliative care, caregiver, quality of life, advanced cancer, thematic analysis

## Abstract

**Introduction:**

Early palliative care (EPC) improves the quality of life (QoL) of advanced cancer patients and their caregivers. The increasingly widespread use of this care model requires the development of measures supporting its interventions. Although the construct of patient’s QoL has been extensively investigated and several QoL measures have been further validated, there is a paucity of data concerning the QoL of the caregiver. In 2018, McDonald and colleagues addressed this issue by interviewing 23 primary caregivers of advanced cancer patients who participated in an EPC randomized clinical trial to understand their perspective on the QoL construct. The Authors identified six major dimensions associated with the construct of caregiver’s QoL. The present retrospective study aimed to validate these dimensions on a larger sample and in a real-life EPC setting.

**Methods:**

Previously collected reports from 137 primary caregivers of advanced cancer patients on EPC answering questions about their experience with this care model were qualitatively analyzed through a deductive, thematic approach to identify and confirm the six dimensions constituting the construct of interest based on McDonald’s and colleagues’ results.

**Results:**

The six dimensions (“living in the patient’s world”, “burden of illness and caregiving”, “assuming the caregiver role”, “renegotiating relationships”, “confronting mortality”, and “maintaining resilience”) were consistently found in the reports from primary caregivers in a real-life EPC setting, confirming to be significant themes associated to their QoL.

**Conclusion:**

A definite and recurrent construct of primary caregiver’s QoL as described by McDonald and colleagues was also found in a larger sample and in a real-life EPC setting. Thus it may lay the groundwork for the development of a dedicated questionnaire.

## 1 Introduction

Early palliative care (EPC) is a recent value-based model providing supportive care for patients who are facing a serious illness, to improve their quality of life (QoL) (1). In the onco-hematology setting, EPC is defined as the early integration of palliative care (PC) into standard care, usually within 8 weeks from the diagnosis of incurable cancer (2–5), in contrast with the model of the standard, late PC, which is usually delivered in the last days of life (6). Yet, EPC and late PC differ in many other aspects beyond just the timing. While, historically, late PC focuses on oncologists or hematologists relieving physical pain at the end of life, EPC also addresses the emotional, social, and spiritual needs of patients over the whole course of the illness through a multidisciplinary team-based approach. According to the EPC model conceptualized by Jacobsen and colleagues (7), this paradigm is based on seven key elements that the palliativist must consider in the patient’s care journey: building rapport, managing symptoms, promoting adaptive coping, developing prognostic awareness, planning advanced care, facilitating treatment decisions, and guiding disposition. These seven elements are to be addressed by the palliativist while accompanying the patients through the five challenges they will encounter: adapting to the diagnosis, pairing hopes and worries, living well with a serious illness, deepening prognostic awareness, and acknowledging the end of life.

One of the distinctive features of the EPC paradigm is the inclusion of the patient’s family and caregiver in the care model. It is widely recognized that the absence of a caregiver negatively impacts a patient’s well-being, treatment adherence, and survival rate, as well as health care costs (8). On the other hand, the presence of a caregiver with a negative performance status may be even more detrimental (9). First, negative caregiver performance may limit the patient’s optimal care and well-being (8, 10). Furthermore, because the demands of caregiving can be overwhelming and jeopardize both physical and emotional resources (11), the caregiver may become what has been defined as a ‘second-order patient’ (8).

The caregiver burden can be present at any stage of the disease trajectory, but its entity and impact on the caregiver’s QoL may vary depending on the stage of the disease. In the early stages, the immediate reaction to the diagnosis often leads caregivers to experience traumatic stress-associated symptoms, identifiable as an acute stress disorder or a post-traumatic stress disorder, leading to an increased risk of cardiovascular, metabolic, and musculoskeletal issues as well as suicide (8, 12–18). As the disease progresses, caregivers must provide more intensive care, manage multiple medications and treatments, and navigate difficult decisions about the end of life (13–15). In these stages, the burden of caregivers can lead them to experience a distress condition equal to or even higher than those experienced by the patients themselves (19). Similar levels of distress have been associated with poor physical health (10, 20, 21) and a higher risk of mortality (22, 23). Moreover, as the terminal phase approaches, caregivers may also be coping with grief and anticipatory mourning (8, 12–18). Furthermore, it should be noted that the caregivers’ emotional load does not end with the patient’s death. Indeed, the literature describes the subsequent period of mourning as characterized by an initial feeling of relief, rapidly replaced by the appearance of stress-related psychological disorders such as anxiety and depression, which negatively affect not only physical health (2, 24), as demonstrated by the increase in drug consumption in this population (25), but compromises social relationships, professional setting, and, more generally, daily routines in a dysfunctional way. It is noteworthy that both the early stage and the mourning stage are neglected by the late PC model.

EPC is the first model of care that has explicitly formalized the inclusion of the caregiver’s QoL into the paradigm, as also recommended by WHO guidelines (26), since the time of the diagnosis and after the patient’s death. As a consequence, the EPC, compared to the late PC, translates into a better output for the caregiver, as, has recently been demonstrated (for reviews, see 27, 28). Notwithstanding, the construct of QoL from the caregiver’s perspective has not been sufficiently investigated and there is paucity of validated measures in this area. In 2002, Edwards and Ung (29) reviewed the QoL measures for caregivers of cancer patients published from 1980 to 2000 and identified 4 scales: the Caregiver Quality of Life Index - Cancer Scale, the Caregiver Quality of Life Index, the Quality of Life Tool, and the Quality of Life Index - Cancer Version. In addition to these, Cohen and colleagues (30) developed a similar questionnaire in 2006, which, however, was specific for the late-stage disease (31). Considering that recent and more effective symptom control methodologies are modifying the disease trajectory by extending the patient’s terminal phase and consequently the caregiving activity, increasing the physical and emotional burden required by caregivers without preparing them for future bereavement (24, 32), these scales may no longer be adequate to reflect aspects related to the construct of caregiver’s QoL.

Before developing a measure for assessing a construct, it is necessary to identify the dimensions that constitute it. This refers to the construct or content validity of the measure, i.e., the extent to which it accurately represents the construct of interest and measures what it claims to measure (33). Qualitative studies, e.g., interviews and questionnaires, provide in-depth insights and understanding of the content, process, and dynamics (i.e., dimensions) of a construct from the perspective of the stakeholder. They also provide a solid foundation to develop the initial pool of items for the measure, ensuring that they are indeed relevant to the target group (34, 35).

Recently, the construct of caregiver’s QoL has been revised by McDonald and colleagues (31), who interviewed 23 primary caregivers who had previously participated in a 4-month EPC randomized clinical trial (14 in the EPC arm and 9 in the standard oncology care arm) to discuss their QoL. Based on the Authors’ results, the construct of caregiver’s QoL is composed of six major dimensions: “living in the patient’s world”, “burden of illness and caregiving”, “assuming the caregiver role”, “renegotiating relationships”, “confronting mortality”, “maintaining resilience”. Of these dimensions, two were not identified in the interviews of caregivers from the standard oncology care arm, and some were underrepresented in existing caregiver QoL scales. This revised construct of caregiver’s QoL lays the groundwork for developing a measure to assess it.

The present retrospective study aims to validate the revised construct of primary caregiver’s QoL as defined by McDonald and colleagues (31), overcoming the limits represented by their small sample size (N = 14) and their short-term, highly controlled setting of EPC (4-month cluster randomized trial), to increase the construct validity of a future measure. To address this issue, we qualitatively analyzed pre-existing reports from 137 primary caregivers of advanced cancer patients in a real-life setting of EPC answering questions about their experience with this care, to look for the presence of themes related to the dimensions of the revised construct of caregiver’s QoL (31).

## 2 Materials and methods

### 2.1 Setting

The provision of EPC described in this work takes place in two Italian EPC units. The first is located at the Oncology and Palliative Care Unit of the Civil Hospital in Carpi, within the Local Health Unit in Modena; the second, at the EPC clinic of the section of Hematology, Azienda Ospedaliero Universitaria Policlinico, University of Modena and Reggio Emilia. The unit in Carpi typically admits patients who are in advanced stages of solid cancer, which includes distant metastases, late-stage disease, and/or prognosis of 6-24 months. Patients at unit in Modena are mainly diagnosed with acute myeloid leukemia or multiple myeloma, although patients with other high-risk hematologic malignancies also receive EPC. In both cases, the intervention is considered “early” if it is provided within eight weeks of the cancer diagnosis and goes on till the bereavement phase. The EPC program implemented in these units integrate primary oncology and hematology specialists with a palliative care team involving doctors, nurses, and eventually social workers and others who work together to provide comprehensive care. Through the establishment of an honest and trusting relationship, the EPC team provides comprehensive symptom management; promotes illness understanding and prognostic awareness; supports patient and caregiver engagement in goals-of-care definition and treatment decisions making, including advanced care planning; facilitates coping; and monitors the family bereavement process through post-death family meetings. These interventions are provided through regular consultations with oncologists/hematologists and nurses (1).

### 2.2 Participants

A total of 137 primary caregivers on EPC were recruited between July 2020 and February 2023 to answer questions on their experience with this model of care. All have cared for or are still caring for a family member with advanced/metastatic cancer diagnosis. Caregiver eligibility required willingness to complete the questionnaire and age ≥ 18 years. All participants provided written informed consent prior to data collection.

The study was performed in accordance with the ethical standards of the 2013 Declaration of Helsinki and was approved by the Ethics Committee of Modena (N. 0026448/20).

### 2.3 Data collection

In the context of a larger research project aiming at investigating the perception of EPC and its possibly related benefits by patients and caregivers, a questionnaire composed of questions exploring the experience with EPC was administered to caregivers. The EPC team in both Carpi and Modena arranged individual, face-to-face meetings with primary caregivers attending the units to provide an explanation of the project and to address clarification requests or concerns about it. Once written consent was obtained, participants were given a written copy of the questionnaire (**Table 1**) with the request to return it within one month. To ensure that respondents could answer the questions comfortably and to minimize the risk of social desirability bias in their answers, a self-administered, anonymous questionnaire was chosen over an oral interview. The questionnaire answers have already been analyzed to gather information on the caregiver’s perception of the disease before and after an EPC intervention (36), the caregiver’s perception of death and hope (37, 38), and the caregiver’s feeling of gratitude (39). For this retrospective study, the answers were reanalyzed.

**Table 1:** Analyzed questions from the questionnaires.

	Questions
1.	*For how long did your relative come to the EPC Unit?*
2.	*What do you think EPC treatments meant for your loved one?*
2.1	*And what did they mean to you?*
4.1	*What do you think is EPC’s role in the treatment of oncologic illness?*
5.	*Do you think EPC treatments allow keeping hope alive?*
5.1	*What is hope for you?*
5.2	*Is there an episode you would like to share with us from the period of time when you were caring for your loved one?*
6.	*Would you like to add something else?*

### 2.4 Data analysis

As we aimed to explore the presence in caregivers’ answers of a construct as defined by a pre-existing theory (31), a deductive approach to thematic analysis was adopted. Specifically, the deductive thematic analysis involved the identification of themes based on the theoretical framework of our interest and its testing against the data. In our case, the themes were already identified and constituted by the six dimensions of the revised construct of caregiver’s QoL (31).

The returned questionnaires had previously been computer-transcribed *verbatim*. Two co-authors (EBa and EBo) independently conducted the analysis. Initially, the transcripts were read repeatedly to become familiar with their contents. Through a gradual, line-by-line coding process, the themes related to the six dimensions of the construct of caregiver’s QoL (“living in patient’s world”, “burden of illness and caregiving”, “assuming the caregiver role”, renegotiating relationships”, “confronting mortality”, and “maintaining resilience”) were identified and assigned. To ensure trustworthiness in the analytic process, ongoing meetings between EBa and EBo were held to discuss and justify the identified themes and their assignment to the six dimensions.

In the Results section, the percentage of responses referring to each dimension has been provided. Additionally, each dimension is supported by illustrative quotations from the caregivers’ responses. These illustrative quotations are reported with the caregivers’ age range and their relationship to patients.

## 3 Results

### 3.1 Sample description

The study sample was composed of 63.5% women and 34.3% men of different ages (age mean: 56.6 years; SD: 13.8; range: 20-82), predominantly Caucasian and Catholic and with different education levels. Demographic and caregiving characteristics of the sample are reported in **Table 2**.

**Table 2:** Demographic and caregiving characteristics of the sample.

			Primary caregivers (n = 137)
Age at questionnaire administration	Years	Mean (sd)	56.6 (13.8)
		Range	20-82
Age group	18-30	n (%)	5 (3.7)
	31-40	n (%)	11 (8)
	41-50	n (%)	33 (24.1)
	51-60	n (%)	31 (22.6)
	61-70	n (%)	30 (21.9)
	71-82	n (%)	22 (16.1)
	Missing data	n (%)	5 (3.7)
Sex	Female	n (%)	87 (63.5)
	Male	n (%)	47 (34.3)
	Missing data	n (%)	3 (2.2)
Education	Primary school	n (%)	10 (7.3)
	Secondary school	n (%)	26 (19)
	College	n (%)	52 (38)
	Graduation’s degree	n (%)	4 (2.9)
	Bachelor’s degree	n (%)	37 (27)
	Missing data	n (%)	8 (5.8)
Ethnicity	Caucasian	n (%)	124 (90.5)
	Arabian	n (%)	2 (1.5)
	Indo-European	n (%)	1 (0.7)
	African	n (%)	1 (0.7)
	Missing data	n (%)	9 (6.6)
Religion	Catholic	n (%)	99 (72.3)
	Muslim	n (%)	2 (1.5)
	Evangelic	n (%)	1 (0.7)
	Orthodox	n (%)	3 (2.2)
	Jehovah’s Witness	n (%)	1 (0.7)
	Agnostic/Atheist	n (%)	21 (15.3)
	Animist	n (%)	2 (1.5)
	Missing data	n (%)	8 (5.8)
Cancer diagnosis	Solid	n (%)	108 (78.8)
	Hematologic	n (%)	29 (21.2)
Time since first EPC consult	Months	Mean (sd)	14.2 (15)
		Range	2-72
Relationship to patient	Spouse/partner	n (%)	61 (44.5)
	Daughter/son	n (%)	61 (44.5)
	Parent	n (%)	1 (0.7)
	Sister/brother	n (%)	4 (2.9)
	Other family	n (%)	5 (3.6)
	Missing data	n (%)	5 (3.6)
Status of the patient at the moment of the caregiver enrollment	Alive	n (%)	100 (73%)
	Deceased	n (%)	37 (27%)
In case of deceased patient, time since death	Months	Mean (sd)	13.4 (10.1)
		Range	1-36

n, number; sd, standard deviation; EPC, early palliative care.

### 3.2 Thematic analysis: themes and quotes

Themes related to the six dimensions of the construct of caregiver’s QoL (“living in patient’s world”, “burden of illness and caregiving”, “assuming the caregiver role”, renegotiating relationships”, “confronting mortality”, and “maintaining resilience”) were identified in all collected answers.

#### 3.2.1 Living in the patient’s world

The dimension “living in the patient’s world” was identifiable in 37% of answers. Caregivers talked about living “in symbiosis” with the patient

*Since my wife became ill, I am living in symbiosis with her suffering and improvements. (Husband, 51-60yrs)*

and they repeatedly reported that what makes the patient feel bad or good, makes them feel bad or good too.

… *psychologically I suffer and rejoice like her according to the outcome of the situation. (Husband, 51-60yrs)*

… *what is good for my husband, is good for me too. (Wife, 61-70yrs)*

The caregiver’s QoL was dependent on the patient’s QoL.

*I could no longer see her suffering like this, I was desperate. (Husband, 51-60yrs)*

… *to see her smile now, without pain, is a salvation for me. (Husband, 51-60yrs)*

In this regard, some caregivers explicitly referred to the role of the EPC intervention in improving the patient’s QoL and, consequently, their own QoL.

*To me, the palliative care has been even more precious; seeing my daughter at peace was like reliving for me and I started smiling again*. *(Wife, 71-82yrs)*

*The palliative care is a holy thing, my wife hasn’t suffered since and what’s more beautiful than that? (Sister, 71-82yrs)*

*Without these palliative cares my sister was desperate and so was I. (Husband, 71-82yrs)*

Overall, the wellbeing of the patient was referred to as a priority over everything else.

*The most important thing for me was that she didn’t suffer. (Daughter, 51-60yrs)*

*… the only thing that matters to me is to see my father without pain (…). (Son, 18-30yrs)*

*To see the dearest person in the world not in pain is really everything. (Husband, 71-82yrs)*

#### 3.2.2 The burden of the illness and caregiving

The dimension “the burden of the illness and caregiving” was identifiable in 37% of answers. Caregivers mentioned the uncertainty associated with the new role

*I found myself catapulted into a world that was unknown to me, (…), so I was almost unaware of what the disease entails and how a person could face the path (…). (Husband, 41-50yrs)*

… *when we were on oncology standard care, no one spoke to me and explained what was going on to me. I was always worried and anxious. (Husband, 71-82yrs)*

as well as the following emotional burden and its consequences.

*Seeing the closest person in this grip of suffering was such a big blow for me that I couldn’t accept it*. *(Husband, 51-60yrs)*

*In 2013 when she began her cancer diagnosis ordeal, I became depressed for a long time, I was even unable to get out of bed. (Husband, 51-60yrs)*

A recurrent theme was associated with the feeling of helplessness experienced by the caregiver when taking care of the patient.

… *he couldn’t even sleep from the pain (…) he couldn’t even eat (…) I felt helpless, not being able to do anything for him. (Wife, 71-82yrs)*

Most of the caregivers explicitly highlighted how EPC was able to reverse the burden of illness and caregiving.

*In this unit I feel taken by hand, I don’t feel alone and desperate as I was when I first came here. I think I could never face such an extreme and hard path without this support. (Wife, 51-60yrs)*

*As a family member, even just a call with the team, made me feel relieved, allowing me to accept the weight of my role. (Husband, 71-82yrs)*

No references to physical, social, and financial distress were found in the transcripts.

#### 3.2.3 Assuming the caregiver role

The dimension “assuming the caregiver role” was identifiable in 32% of answers. Several transcripts mentioned how EPC facilitated the transition to the role of caregiver.

*EPC provides great support, to see my mother without pain and serene allows me to accompany her as a caregiver (beyond a doctor) in the calmest way possible. (Daughter, 51-60yrs)*

*Seeing my husband feeling better and, even more, without suffering has helped me a lot in fulfilling my role of caregiver. Without the EPC I would certainly not have been able to face this long and burdensome journey. (Wife, 61-70yrs)*

*Now I feel able to take care of her, to be close to her. To be helpful. (Husband, 51-60yrs)*

Most participants explicitly referred to the honest conversations with the EPC team as pivotal in facilitating the switch in the caregiver role.

*EPC are more and more fundamental: knowing what is happening, knowing the prognosis of the disease, being able to talk to the doctors about possible choices … This makes me more aware, more prepared, and allows me to be able to follow my mother in the best possible way. (Daughter, 41-50yrs)*

*Knowing made me aware and this and only this has allowed me to prepare myself and my son and is allowing me to be able to support my husband. (Wife, 41-50yrs)*

The presence of both the patient and the caregiver during these encounters had a key role.

*The interviews I had with the doctor in the presence of my dad were very helpful. (Husband, 51-60yrs)*

Specifically, honest conversations facilitated the provision of emotional support as well as advocacy.

*EPC was fundamental, because the conversations I had with the doctor provided me with all the information on the real clinical situation and prognosis, supporting me in taking the best decisions; for example, for me, it was essential to talk to the palliative doctor to decide in complete serenity to suspend yet another chemotherapy which was no longer needed and which my father no longer tolerated. (Daughter, 41-50yrs)*

*Two things were fundamental: to see my father free from physical pain and to have periodic conversations with the doctor, who was able to accompany me in understanding the real situation, giving me the tools to know what to do time by time and giving me the awareness that I would never be abandoned to myself. (Daughter, 41-50yrs)*

A participant described how assuming the caregiver role would concretely not possible without EPC.

*The presence of beloved ones has a strong and essential role in support and comfort, but unfortunately, it is not enough. I can guarantee my presence and my support to my wife, but I can’t give her medically reliable answers, I can’t relieve her pains, her symptoms; this is why EPC is needed. (Husband, 51-60yrs)*

#### 3.2.4 Renegotiating relationships

The dimension “renegotiating relationships” was identifiable in 12% of answers. A few caregivers reported a patient’s emotional burnout affecting the relationship.

*My husband withdrew into himself and could no longer speak. (Wife, 31-40yrs)*

*At first, my father didn’t want to talk to us anymore* (…). *(Daughter, 51-60yrs)*

However, EPC intervention was able to restore it.

*… now she is fine, she is no longer in pain and can lead a life as a mother and wife as before. (Husband, 51-60yrs)*

Caregivers also referred to having had conversations with the patient requiring a deep bond.

*I pondered with my father. (Son, 18-30yrs)*

*The interviews I had with the doctor in the presence of my mother were very helpful. My mom is better than me and more protective. (Husband, 51-60yrs)*.

#### 3.2.5 Confronting mortality

The dimension “confronting mortality” was identifiable in 61% of answers. Caregivers talked openly about the future and the prognosis mentioning the conversations with the EPC team as having a facilitating role in facing the conversation.

*I must say that the conversations with the doctor about my mother’s illness and conditions are paradoxically a source of serenity and not a source of anguish. Before, when I didn’t have the opportunity to talk to the oncologists, I felt anxious and full of fear. To me, knowing the truth was fundamental to being able to accept the idea of ​​death. While before I believe that the illusion prevailed. (Daughter, 51-60yrs)*

*Two things were fundamental, seeing my father free from physical suffering and having repeated talks with a doctor who was able to accompany me in understanding the truth of the situation, giving me the tools to know what to do every time and knowing that I would never be abandoned to myself. (Daughter, 41-50yrs)*

When talking about the future, caregivers focused on the importance of a painless death,

*Now I know that pain can be managed and I am calmer and have less fear of death. (Wife, 41-50yrs)*

*Death is a natural but terrible thing if it happened with devastating pains and when one feels abandoned and desperate. This is true for the patient but also for the family member. (Wife, 61-70yrs)*

knowing the truth,

*Knowing the prognosis is helping me in dealing with my husband’s death. (Wife, 41-50yrs)*

being able to honestly talk about it,

*We were very scared; we didn’t know how to approach the topic with him. (Son, 51-60yrs)*

*We are much more serene because we have been able to talk about it, while doctors, in general, don’t do that, they are scared, I think, and not prepared. (Daughter, 18-30yrs)*

and being supported by someone else.

*I feel I am not alone in facing death and this allows me to be able to manage it, to be close to my aunt who has no one but me. Previously, I felt alone and I think it is the loneliness that does not allow to accept death. (Nephew, 61-70yrs)*

#### 3.2.6 Maintaining resilience

The dimension “maintaining resilience” was identifiable in 15% of answers. Caregivers highlighted how EPC allowed them to cope with the situation. They mentioned strategies like focusing on having a good QoL, and enjoying family,

*Palliative cares are allowing me, my wife and our daughter to keep the focus on the quality of life and living life as a family, one day at a time* (…) *My and my wife’s first thought was: ok, no one has died yet. Our second thought was: what can we do to live well? Then, we started to roll up our sleeves. In the family, you can feel that something has changed, but this does not mean that we have to cancel our projects, they are just undergoing a slowdown and some changes. (Husband, 41-50yrs)*

living one day at a time.

*Of course, we all live one day at a time dealing with problems as they arise*. *(Daughter, 41-50yrs)*

One caregiver reported having named the disease.

*Me as a family member and my wife as well ​​have the opportunity to focus on the real situation and, while doing so, to fight on an even footing, in fact, we are a small army, while “Estore” (the disease) is alone. (Husband, 41-50yrs)*

Maintaining resilience was facilitated by some behaviors from the EPC team, like treating the caregiver as a person

*It was essential for me to feel welcomed as a human and listened to in my needs. (Daughter, 51-60yrs)*

and being available to confront.

*I must say that the conversations with the doctor about my mother’s illness and conditions are paradoxically a source of serenity and not a source of anguish. (Daughter, 51-60yrs)*

*Two things were fundamental, (…) and having repeated conversations with a doctor who was able to accompany me in understanding the truth of the situation, giving me the tools to know what to do every time and knowing that I would never be abandoned to myself. (Daughter, 41-50yrs)*

## 4 Discussion

The present retrospective study sought to validate the construct of QoL from the primary caregiver’s perspective as described by McDonald and colleagues (31), by identifying themes related to its constructs in a sample of 137 primary caregivers of advanced cancer patients talking about their experience with EPC in a real-life setting. Overall, our results confirmed the validity of the construct, by identifying recurrent themes associated with the dimensions of the revised construct of caregiver’s QoL, although the percentages of responses referring to each of them should be interpreted with caution, as it may be biased by the nature of questions included in the original questionnaire. At the same time, we found some differences that can be attributed to the experience of EPC in a real-life setting.

The dimension “Living in the patient’s world” refers to a switch of the focus from the self to the patient, to prioritize the patient’s preferences, values, and goals when providing care. This dimension represented a core theme mentioned by almost all of our caregivers, as found also by McDonald and colleagues (31), and is supported by previous quantitative studies, highlighting an association between the caregiver’s QoL and patient wellbeing (14, 40). The significance caregivers attribute to their bond with the patient and the impact of the patient’s well-being on their own QoL highlights the necessity of employing QoL assessments tailored to caregivers, rather than relying on measures designed for broader populations.

McDonald and colleagues observed two different attitudes among caregivers: some of them seemed to experience the change in focus from themselves to the patient as a burden, while others demonstrated greater resilience in accepting it. We also found these two opposing attitudes but reported together, related to a temporal dimension, as a before and after. Specifically, caregivers referred to the refocusing as a burden when talking about their past clinical experience, before the EPC intervention, and as a resilient acceptance when discussing their present clinical experience with the model. This finding can be explained by a longer involvement in EPC care for our participants, averaging 14 months, compared to the shorter involvement of McDonald and colleagues’ participants, which was of 4 months. This attitude change can be interpreted in the context of a previous study conducted by our group (36). In this study, aimed at ordering the recognized EPC benefits over time, the first EPC intervention identified was assessing and addressing patients’ physical symptoms. Prompt symptom management has been found to have a twofold effect: it restored physical functioning, mood, and social life, allowing patients to return to their previous lives, and it led to a higher availability of psychological resources for coping with the new situation (36). Following the significant reduction of the patient’s disease burden, the caregiver’s perception may change, from being no longer focused on a suffering individual but rather being focused on someone grateful for being back to life, as also found by Borelli and colleagues (39). In this situation, shifting the focus from oneself to the patient becomes less demanding.

We also observed differences concerning “The burden of the illness and caregiving,” a dimension that encompasses the physical, emotional, social, and financial challenges caregivers may face, while taking care of the patient. While McDonald and colleagues found a significant emotional burden, followed by physical, social, and financial distress in their sample of caregivers, our study suggested a different pattern. In our participants, the dimension associated with the burden of this caregiving role appeared to be less pronounced. They mentioned the emotional burden, but not the physical, social, and financial counterparts. Furthermore, we found that themes related to the emotional burden were more commonly referred to their past experiences before the EPC intervention. Overall, participants tended to describe the emotional load as something they can now tolerate, thanks to the support provided by the EPC intervention, but that they would not have been able to handle without it.

The dimension associated with “Assuming the caregiver role” was consistently encountered in our sample’s reports. “Assuming the caregiver role” refers to the caregivers taking on the responsibility of providing both emotional and physical support to the patients, often forcing them to set aside their emotions. Differently from McDonald and colleagues, most of our caregivers mentioned how EPC supported them in the transition to the new role and in managing the associated tasks. Notably, frequent conversations and honest communication with the EPC team played a key role in this transition. Understanding reality facilitated caregivers in assuming their role and providing emotional support.

An important aspect within this dimension was the advocating activity. However, unlike caregivers from McDonald and colleagues, who mainly advocated for their patients, our caregivers extended their advocacy to other patients, emphasizing the importance of spreading the model of EPC to everyone dealing with a cancer diagnosis.

The presence of both the patient and the caregiver during conversations with doctors was crucial in helping the caregiver to address important issues that cannot be neglected while assuming the caregiver role. This process possibly normalized topics often described as “the elephant in the room” (36).

The few existing validated measures in cancer settings do not adequately capture caregivers’ changes in roles and responsibilities, which are consistently found in our reports as well as in those from McDonald and colleagues (41). This may be because several of these measures were developed between 1980 and 1999 (29) and may not reflect current societal norms (e.g., in the employment of women) or the increasing outpatient delivery of cancer treatments (31).

The significant role of frequent and honest conversations with the EPC team was further emphasized when discussing the dimension of “Renegotiating relationships”. The diagnosis of advanced cancer can result in substantial changes in the relationship between the patient and the caregiver. The caregiving role may become more intense, and the patient may become more dependent on the caregiver for physical, emotional, and practical support. However, unlike the findings of McDonald and colleagues, this theme was not frequently mentioned in our reports. It can be speculated that one of the initial benefits of EPC might involve the successful reframing of the relationship between the caregiver and the patient.

“Confronting mortality” is arguably one of the most challenging tasks that caregivers face, beginning even before the patient’s death and often manifesting as anticipatory grief (42). EPC offers several strategies that caregivers can employ to face mortality and manage their emotional distress. These strategies include providing social support and addressing the topic honestly (38).

It is commonly believed that discussions about death should be avoided, both in everyday life and in clinical settings with individuals facing the end of life. However, a recent study (38) challenges this assumption. The study found that advanced cancer patients and caregivers tend to use language related to death more frequently than the general population, suggesting a potential need for more open and honest conversations about the end of life.

Similar to participants in McDonald’s study, our caregivers reported having a realistic perspective on death, where hope remains present but carries a pragmatic meaning. To hope, in their view, means to focus on achieving a good death, free from suffering (37, 38).

Overall, our data confirmed McDonald’s and colleagues’ data, showing that the six major dimensions they identified may be encountered also in a real-life EPC setting and that EPC interventions positively impact them. Thus, a caregiver QoL assessment cannot disregard them.

A limitation of this retrospective study is that the data have not been collected specifically for the research question at hand; thus, they may be subject to bias or errors. On the other hand, a strength of the study consists of the high sample size and the real-life setting, that can increase the generalizability of the findings. In addition to the urgent need for the development of a caregiver’s QoL measurement tool based on the construct definition identified by McDonald and colleagues (31) and validated in our study, an important future direction involves exploring the differences in the construct itself between oncological and onco-hematological patients.

Although far from being the standard of care, the increasingly widespread use of the EPC model makes it critical to define measures supporting its interventions. In this context, the construct of patient’s QoL has been extensively investigated, being one of the most relevant outcomes (43–45), and several measures have been further validated (46–49). Conversely, there is a lack of data concerning the QoL from the primary caregiver’s perspective. Findings from this retrospective study may lay the groundwork for the development of dedicated measures that will improve the benefits of the EPC model.

## Data availability statement

The datasets presented in this article are not readily available because the data underlying this article will be shared on reasonable request to the corresponding author. Requests to access the datasets should be directed to Eleonora Borelli, eleonora.borelli@unimore.it.

## Ethics statement

The studies involving humans were approved by Ethics Committee of Modena (N. 0026448/20). The studies were conducted in accordance with the local legislation and institutional requirements. The participants provided their written informed consent to participate in this study.

## Author contributions

EBa and ML conceptualized the study. EBa, ML and EBo designed the experiment. EBa and LP collected the data. EBa and EBo analyzed the data. EBa, ML and EBo contributed to the interpretation of the results. EBo drafted the manuscript. EBo, SB, LP, FG, FE, CAP, ML and EBa provided critical feedback on the manuscript and contributed to the revisions. EBo, SB, LP, FG, FE, CAP, ML and EBa approved the final version of the manuscript for submission.
